# Supplementary material for: Proteotranscriptomic Profiling of the Toxic Mucus of Kulikovia alborostrata (Pilidiophora, Nemertea)
Source: Toxins (Basel). 2024 Dec 26;17(1):5. doi: 10.3390/toxins17010005 (PMC11768458; doi:10.3390/toxins17010005)
Supplement: Supplementary file 1 [file toxins-17-00005-s001.zip › Supplementary File S4.zip!/ORF 001200 LINALB/1362d454f90accc2/hit_summary.html]

Phyre 2 Results for ORF\_001200\_LINALB

|  |  |
| --- | --- |
| Email | vas9i-kz@mail.ru |
| Description | ORF\_001200\_LINALB |
| Date | Fri Apr 7 03:17:43 BST 2023 || Unique Job ID | 1362d454f90accc2 |

|  |  |
| --- | --- |
| Detailed template information |  |

| # | Template | Alignment Coverage | 3D Model | Confidence | % i.d. | Template Information |
| --- | --- | --- | --- | --- | --- | --- |

|  |  |  |  |  |  |  |
| --- | --- | --- | --- | --- | --- | --- |
| 1 | c2f2jA\_ | --- \| --- \| --- |  | 81.2 | 61 | **PDB header:**antimicrobial protein **Chain:** A: **PDB Molecule:**kalata-b1;  **PDBTitle:** solution structure of [w19k, p20n, v21k]-kalata b1  **PDB Entry:** PDBe RCSB PDBj |
| 2 | d1n1ua\_ | --- \| --- \| --- |  | 67.2 | 61 | **Fold:** Knottins (small inhibitors, toxins, lectins) **Superfamily:** Cyclotides **Family:** Kalata B1 **PDB entry:** PDBe RCSB PDBj |
| 3 | c1n1uA\_ | --- \| --- \| --- |  | 67.2 | 61 | **PDB header:**antibiotic **Chain:** A: **PDB Molecule:**kalata b1;  **PDBTitle:** nmr structure of [ala1,15]kalata b1  **PDB Entry:** PDBe RCSB PDBj |
| 4 | c7k7xA\_ | --- \| --- \| --- |  | 62.5 | 61 | **PDB header:**immunosuppressant **Chain:** A: **PDB Molecule:**cyclotide pase a;  **PDBTitle:** solution structure of the cyclotide pase a  **PDB Entry:** PDBe RCSB PDBj |
| 5 | c4ttoA\_ | --- \| --- \| --- |  | 60.5 | 61 | **PDB header:**plant protein **Chain:** A: **PDB Molecule:**kalata-b1;  **PDBTitle:** quasi-racemic structure of [v25a] kalata b1  **PDB Entry:** PDBe RCSB PDBj |
| 6 | c4ttmA\_ | --- \| --- \| --- |  | 59.2 | 61 | **PDB header:**plant protein **Chain:** A: **PDB Molecule:**kalata-b1;  **PDBTitle:** racemic structure of kalata b1 (kb1)  **PDB Entry:** PDBe RCSB PDBj |
| 7 | d1nb1a\_ | --- \| --- \| --- |  | 59.0 | 61 | **Fold:** Knottins (small inhibitors, toxins, lectins) **Superfamily:** Cyclotides **Family:** Kalata B1 **PDB entry:** PDBe RCSB PDBj |
| 8 | c1nb1A\_ | --- \| --- \| --- |  | 59.0 | 61 | **PDB header:**antibiotic **Chain:** A: **PDB Molecule:**kalata b1;  **PDBTitle:** high resolution solution structure of kalata b1  **PDB Entry:** PDBe RCSB PDBj |
| 9 | c2khbA\_ | --- \| --- \| --- |  | 58.9 | 61 | **PDB header:**antimicrobial protein **Chain:** A: **PDB Molecule:**kalata-b1;  **PDBTitle:** solution structure of linear kalata b1 (loop 6)  **PDB Entry:** PDBe RCSB PDBj |
| 10 | c7lhcA\_ | --- \| --- \| --- |  | 58.0 | 61 | **PDB header:**plant protein **Chain:** A: **PDB Molecule:**kalata-b1;  **PDBTitle:** nmr solution structure of [t20k]kalata b1  **PDB Entry:** PDBe RCSB PDBj |
| 11 | c2mn1A\_ | --- \| --- \| --- |  | 37.7 | 61 | **PDB header:**unknown function **Chain:** A: **PDB Molecule:**kalata b1[w23ww];  **PDBTitle:** solution structure of kalata b1[w23ww]  **PDB Entry:** PDBe RCSB PDBj |
| 12 | c4ttnA\_ | --- \| --- \| --- |  | 57.8 | 57 | **PDB header:**plant protein **Chain:** A: **PDB Molecule:**kalata-b1;  **PDBTitle:** quasi-racemic structure of [g6a]kalata b1  **PDB Entry:** PDBe RCSB PDBj |
| 13 | d1pt4a\_ | --- \| --- \| --- |  | 57.5 | 57 | **Fold:** Knottins (small inhibitors, toxins, lectins) **Superfamily:** Cyclotides **Family:** Kalata B1 **PDB entry:** PDBe RCSB PDBj |
| 14 | c2kukA\_ | --- \| --- \| --- |  | 39.3 | 57 | **PDB header:**antiviral protein **Chain:** A: **PDB Molecule:**leaf cyclotide 2;  **PDBTitle:** solution structure of vhl-2  **PDB Entry:** PDBe RCSB PDBj |
| 15 | c7rfaA\_ | --- \| --- \| --- |  | 35.9 | 59 | **PDB header:**biosynthetic protein **Chain:** A: **PDB Molecule:**kalata-b4;  **PDBTitle:** nmr solution structure of linear [t20k]kalatab1  **PDB Entry:** PDBe RCSB PDBj |
| 16 | c1jjzA\_ | --- \| --- \| --- |  | 35.3 | 59 | **PDB header:**plant protein **Chain:** A: **PDB Molecule:**kalata-b4;  **PDBTitle:** refined structure and disulfide pairing of the kalata b1 peptide  **PDB Entry:** PDBe RCSB PDBj |
| 17 | c1k48A\_ | --- \| --- \| --- |  | 33.4 | 59 | **PDB header:**plant protein **Chain:** A: **PDB Molecule:**kalata b1;  **PDBTitle:** refined structure and disulfide pairing of the kalata b1 peptide  **PDB Entry:** PDBe RCSB PDBj |
| 18 | c2f2iA\_ | --- \| --- \| --- |  | 50.1 | 63 | **PDB header:**antimicrobial protein **Chain:** A: **PDB Molecule:**kalata-b1;  **PDBTitle:** solution structure of [p20d,v21k]-kalata b1  **PDB Entry:** PDBe RCSB PDBj |
| 19 | c2jwmA\_ | --- \| --- \| --- |  | 30.0 | 46 | **PDB header:**plant protein **Chain:** A: **PDB Molecule:**kalata-b7;  **PDBTitle:** nmr spatial srtucture of ternary complex kalata b7/mn2+/dpc micelle  **PDB Entry:** PDBe RCSB PDBj |
| 20 | c3e4hA\_ | --- \| --- \| --- |  | 15.6 | 46 | **PDB header:**plant protein **Chain:** A: **PDB Molecule:**varv peptide f;  **PDBTitle:** crystal structure of the cyclotide varv f  **PDB Entry:** PDBe RCSB PDBj |
| 21 | c2m9oA\_ | --- \| --- \| --- | not modelled | 15.4 | 46 | **PDB header:**plant protein **Chain:** A: **PDB Molecule:**kalata-b7;  **PDBTitle:** solution structure of kalata b7  **PDB Entry:** PDBe RCSB PDBj |
